# Virtual reality-based oculography detects internuclear ophthalmoplegia in multiple sclerosis and other neurological disorders

**DOI:** 10.1007/s00415-026-13906-x

**Published:** 2026-06-06

**Authors:** Emilie Reuter, Justus Luerweg, Melina Katsimpoura, Johanna Haselon, David Bratek, Zacaria Assfoor, Caroline Schuetrumpf, Rafael Klimas, Julia Jessen, Carsten Saft, Ilya Ayzenberg, Anna Lena Fisse, Kalliopi Pitarokoili, Christiane Schneider-Gold, Ralf Gold, Jeremias Motte, Ruth Schneider, Anke Salmen

**Affiliations:** Ruhr University Bochum, St. Josef-Hospital, Department of Neurology, Gudrunstrasse 56, 44791 Bochum, Germany

**Keywords:** INO, MS, Eye-tracking, Oculometrics, Eye movement disorders, Brainstem dysfunction

## Abstract

**Background:**

Clinical–neurological examination may underestimate internuclear ophthalmoplegia (INO). We investigated immersive virtual reality (VR)-based eye-tracking in patients with multiple sclerosis (MS), other neurological disorders (ND), and healthy controls (HC) to detect INO.

**Methods:**

In this prospective monocentric study, 471 participants (243 MS, 104 ND, 124 HC) underwent horizontal prosaccade testing (± 10°, ± 20°) using VR-based oculography. INO was determined in a blinded manner based on software-generated reports comparing adduction and abduction velocities. VR-based and clinical assessments were compared using Cohen’s kappa (κ). Associations with demographic and clinical variables were analyzed using logistic regression.

**Results:**

VR-based assessment identified 43 cases of INO (40 ND, 3 HC). Agreement between VR-based and clinical assessment was moderate (*κ* = 0.42; 92.5% observer agreement; *n* = 414). Three cases were identified clinically only, whereas 28 were detected by VR-based assessment only.

Within MS, agreement was similar (*κ* = 0.45; 89.1% agreement; *n* = 238), with 23 cases detected by VR only and three clinically only. In MS, VR-based INO was associated with male sex (*p* = 0.035), higher expanded disability status scale (EDSS) and brainstem functional system scores (*p* < 0.001), longer disease duration and older age (*p* = 0.002), and visual complaints (*p* = 0.007). In multivariable analysis, disease duration (OR 1.12, 95% CI 1.01–1.24), brainstem involvement (OR 21.59, 1.30–358.07), and clinical INO (OR 8.18, 1.43–46.96) were associated with VR-based INO.

**Conclusions:**

VR-based eye-tracking showed moderate agreement with clinical assessment and identified additional cases of INO associated with markers of disease burden. These findings support further evaluation of VR-derived measures as complementary digital biomarkers of brainstem dysfunction.

**Supplementary Information:**

The online version contains supplementary material available at 10.1007/s00415-026-13906-x.

## Introduction

Internuclear ophthalmoplegia (INO) constitutes the most frequent oculomotor dysfunction in multiple sclerosis (MS) with a prevalence of 15–52% and can occur uni- or bilateral [1–4]. The eye movement disorder can present acutely during MS relapse and chronically during continuous progression of MS [5]. People with INO often experience diplopia (35,2%), dizziness (42–48%), and nystagmus of the abducting eye (up to 44%) deteriorating their quality of life [1, 5]. INO is defined by dysconjugation of the horizontal eye movement. It shows a relative slowing of the adduction of the eye ipsilateral of the lesion over the abduction of the contralateral eye often accompanied by dissociated horizontal jerk nystagmus of the contralateral eye [6]. The lesion localization is typically in the medial longitudinal fasciculus (MLF) [1], a nerve bundle located in the pons and mesencephalon, ventral to the fourth ventricle and the cerebral aqueduct [7, 8]. Coordination of horizontal eye movement is achieved by connecting the ipsilateral oculomotor nucleus of the adducting eye with the contralateral abducens nucleus [1, 7–9]. Lesions in the MLF disrupt this pathway and can result from inflammatory, vascular, infectious, or traumatic causes [9, 10]. Lesions are often missed in Magnetic Resonance Imaging (MRI) with a sensitivity of only 46,2%, potentially resulting from limited resolution of MRI techniques, especially for small lesions [6]. This may result in a delay in the provision of appropriate treatment of symptoms and the underlying cause. Prompt diagnosis is essential to improve outcome and regain a higher quality of life [1], can prevent long-term sequelae [11] and can support differentiation of MS and Neuromyelitis Optica spectrum disorder (NMOSD) [12].

INO is clinically identified by examining eye movements during horizontal gaze, focusing on the discrepancy between the velocities of adduction of one eye and abduction of the other eye [5]. In mild INO, a marginal discrepancy is frequently overlooked, especially in the absence of nystagmus [13]. For objective and more precise diagnosis, infrared oculography (IRO) and Video-Oculography (VOG) can be applied [1, 6]. The ratio between abduction and adduction measured by IRO can be expressed through the versional dysconjugacy index (VDI). This allows objective and sensitive comparison of different forms of INO and documentation of INO evolution over time [1]. Both IRO and VOG depict valid approaches to objectify INO. Neos^®^/PRET™ (machineMD, Bern, Switzerland) is a medical device based on a commercially available VR-based headset running a standardized, automated program, delivering (among other parameters) measurements of the eye movements in all directions of gaze [14]. The setup allows for bedside testing and variation in head position. The intuitive visualization for the examined person facilitates compliance. Focusing on horizontal saccades, we aimed to objectively determine INO using an easy blinded evaluation algorithm based on simple visual inspection of the provided standardized examination report as a potential functional biomarker of brainstem involvement in MS and other neurological disorders.

## Methods

### Standard protocol approvals, registrations, and patient consents

The study was approved by the ethics committee of the Medical Faculty of Ruhr University Bochum, Bochum, Germany (approval number 23–7934-BR) and conducted in accordance with the ethical standards laid down in the 1964 Declaration of Helsinki and its later amendments. Written informed consent was obtained from all participants.

### Study design and patient population

The prospective observational exploratory study was carried out monocentrically from 11/2023 to 01/2025 at the Department of Neurology, St. Josef-Hospital, Ruhr-University Bochum.

Inclusion criteria for all participants comprised a minimum age of 18 years. For patients, a confirmed diagnosis of a neurological disorder under routine neurological care (in- or outpatient setting) at the site was required. For healthy controls, eligibility required the absence of any known neurological condition and willingness to undergo a one-time clinical and VR-based examination.

Exclusion criteria were lacking capacity to provide informed consent, and individuals with regular or intermittent recent use of harmful substances (e.g., alcohol, psychoactive drugs with sedative or stimulant properties, benzodiazepines, phenylethylamines, amphetamine derivatives).

### Clinical history and assessment

As part of the participants’ medical history assessment, data were collected on age, sex, the presence of a neurological disorder, the duration of the disorder (if applicable), current medication use, and any subjective visual complaints as well as disease-specific additional information (e.g., relapse, antibody status, disease-specific scores).

The clinical examination included a comprehensive assessment of neurological status, with a particular focus on neuro-ophthalmological function. This encompassed the evaluation of pupillary motility, visual acuity—using corrected near vision and/or high- and low-contrast acuity tests via Sloan letter charts, visual field testing (finger perimetry), and the manual assessment of smooth pursuit and saccadic eye movements. The test of skew, the head impulse test, and a general inspection of ocular alignment, head posture, and external eye appearance were performed. The examinations were performed in a standardized manner after repeated training of all raters (training performed by AS (senior physician), raters were residents or graduate students). The clinical examination was conducted independently and prior to the VR assessment and its later evaluation for INO.

To quantify the degree of disability in patients with MS, NMOSD, and Myelin Oligodendrocyte Glycoprotein Antibody-Associated Disease (MOGAD), the current Expanded Disability Status Scale (EDSS) score was determined [15]. All patients were diagnosed according to the current diagnostic criteria at inclusion [16–18].

### Magnetic resonance imaging

Available brain MRI scans from clinical routine were re-assessed by an MRI-trained neurologist for all participants with clinical and/or VR-based detection of INO for potential detection of a corresponding lesion. MRI assessment thus served as a targeted confirmatory test.

### Virtual reality (VR)-based oculography

For VR-based oculography, the neos®/PRET™ system (machineMD, Bern, Switzerland) generates visual stimuli on the LED display of the Varjo VR headset. Eye and pupil movements are recorded using an integrated infrared sensor operating at a frequency of 200 Hz.

Horizontal saccadic eye movements are measured five times between ± 10° and four times between ± 20° with a binocular stimulus. Additional information regarding the technical setup can be found in the Supplementary Methods.

**Fig. 1:**
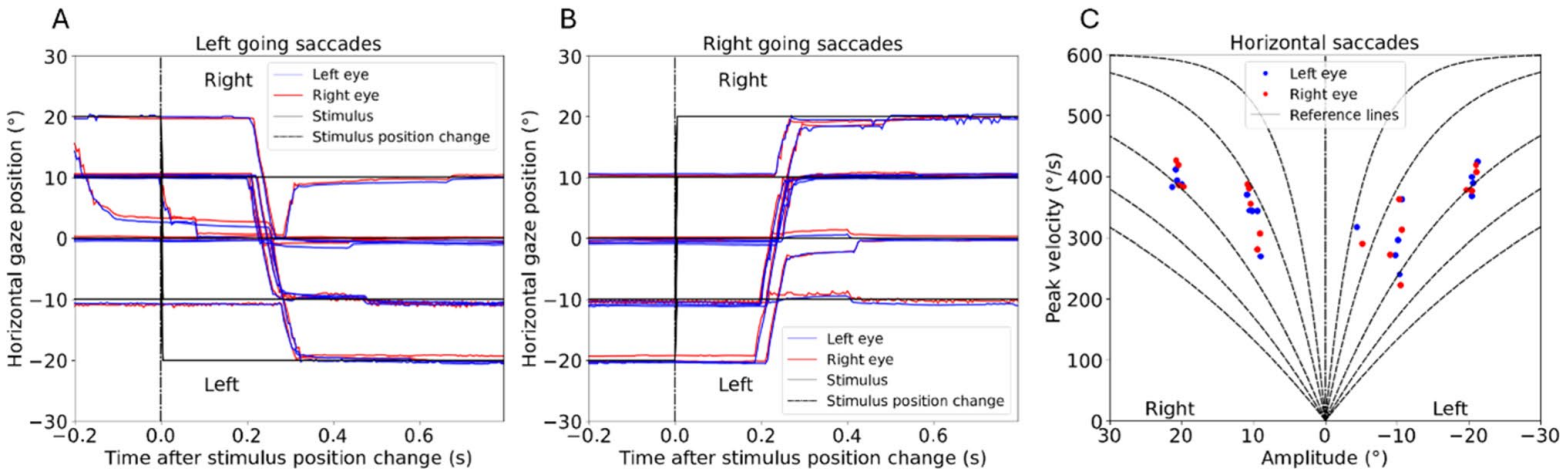
Representation of saccade testing in the standardized VR-report. **A**, **B** Left/right going saccades of the right (red) and left (blue) eye after presentation of the stimulus and its change in position (black line). **C** Diagram illustrating maximum saccadic velocities of the measured values for the right (red) and left (blue) eye at amplitudes of ± 10° and ± 20° of a healthy participant. For reference, the diagram includes the logarithmic representation of the saccadic “main sequence”, which characterizes the amplitude-dependent increase in saccade velocity [19]. The latter represent the normative range of the saccadic “main sequence”. Data points within this fan-shaped area fall within physiological variability, whereas systematic deviations indicate abnormally (usually slowed) saccades

Upon completion of the examination, the software automatically generates a report containing the recorded data (Fig. 1).

### INO detection

**Fig. 2:**
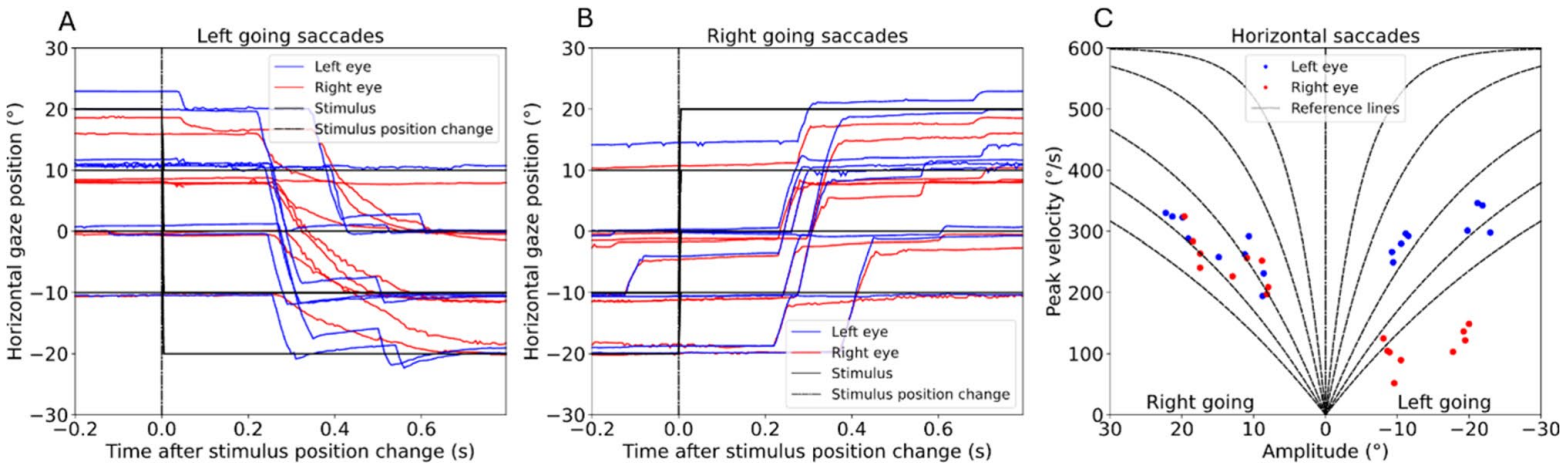
Example of unilateral internuclear ophthalmoplegia (INO). **A**, **B** Left/right going saccades after presentation of the stimulus. In figure A, a delayed adduction of the right eye (red) compared to the left eye (blue) is observed following a change in stimulus position. **C** Maximum saccadic velocities of the measured values for the right (red) and left (blue) eye at amplitudes of ± 10° and ± 20° of a unilateral INO to the right. The peak velocities of the right eye (red) are systematically reduced during leftward gaze compared to the left eye (blue)

INO was defined as a relative slowing of adduction compared to abduction, as observed in the diagram of maximum horizontal saccade velocities (Figs. 1, 2). Absolute peak velocities were not taken into account. INO was defined present when the majority of measured adducting saccades for both the 10° and 20° stimuli were visibly below the abducting saccades (Fig. 2C, left going saccades) and not present in case of overlapping ad- and abducting peak velocity values (Fig. 2C, right going saccades). INO was then categorized as “no INO,” “unilateral INO” (with side specification), or “bilateral INO”. The evaluation of the reports for the presence of INO was performed independently and in a blinded manner by two assessors (JL, resident; ER, graduate student). In case of disagreement between the two evaluations, a third blinded reviewer judged the report (AS, senior physician) and the majority answer (2 of 3) was included.

### Statistical analyses

Descriptive statistics were used to characterize the study population, with more detailed analyses performed for the subgroup of patients with MS. VR-based INO findings were compared with clinical assessment using Cohen’s kappa coefficient and observer agreement rates to quantify inter-method agreement, as no independent gold standard for INO was available and clinical examination was not assumed to represent a definitive reference. Group comparisons were performed using Chi-square/Fisher’s exact test or Mann–Whitney *U* test, respectively.

Binary logistic regression was performed to explore relationships of VR-based INO and clinical variables, with presence of VR-based INO as the dependent variable. All variables identified as significant within the single variable testing were entered into the model as independent variables.

With the MRI as a targeted confirmatory test, conventional diagnostic accuracy metrics were prone to verification bias. We therefore used conditional confirmation probabilities (P(MRI + │VR +); P(MRI + │VR-); P(MRI + │clinical +); P(MRI + │clinical-)).

A significance level of *p* < 0.05 was applied to all statistical tests. Adjustment of *p* values for multiple testing was not performed as all variables with *p* < 0.05 entered the final regression model thereafter.

All analyses were conducted using IBM SPSS Statistics Version 30.0 for Windows (IBM Corp. (2024). Graphical representation of the regression model was designed using GraphPad Prism Version 10.6 for Windows (GraphPad Software, LLC).

### Data availability

Anonymized data may be shared upon request by qualified investigators via the corresponding author respecting regulatory requirements with regards to data protection. Data underlying these analyses are based on blinded rating implemented as a simple present/not-present coding and thus in a tabular format. Relevant sections of the generated report are in pdf format. Raw data underlying the measurements were not used in this study.

## Results

### Characteristics of the whole and the MS cohort

Of 540 VR-based oculography examinations in total, 4 (0.7%) were terminated due to dizziness. From these examinations, 3 were MS (*n* = 2 relapsing remitting [RR], *n* = 1 secondary progressive [SP]) and 1 Huntington’s Disease (HD). All available baseline examinations (*n* = 471 in total; n = 124 HC, *n* = 347 ND) were evaluated (Table 1).

**Table 1:** Cohort characteristics of healthy controls, multiple sclerosis spectrum and other neurological disorders

	Sex, female n (%)	Age, y, mean (SD)	HCVA, mean (SD)		LCVA, mean (SD)		EDSS, median (IQR)	FS brainstem, *n* (%)					Time from onset, y, mean (SD)	Time since diagnosis, y, mean (SD)
			right eye	left eye	right eye	left eye		0	1	2	3	4		
Healthy controls *n* = 124	71 (57.3)	42.46 (± 16.43)	1.01 (± 0.29) (available for *n* = 98)	1.03 (± 0.3) (available for *n* = 97)	0.37 (± 0.14) (available for *n* = 67)	0.37 (± 0.12) (available for *n* = 67)								
MS-spectrum *n* = 243	164 (67.5)	42.73 (± 12.72)	0.95 (± 0.31) (available for *n* = 168)	0.96 (± 0.33) (available for *n* = 170)	0.31 (± 0.18) (available for *n* = 120)	0.3 (± 0.18) (available for *n* = 123)	2.0 (2.0) (available for *n* = 229)	121/209 (57.9)	52/209 (24.9)	25/209 (12.0)	9/209 (4.3)	2/209 (1.0)	11.85 (± 9.85) (available for *n* = 225)	10.33 (± 9.34) (available for *n* = 233)
RIS *n* = 18	10 (55.6)	32.85 (± 7.66)	1.17 (± 0.27) (available for *n* = 12)	1.17 (± 0.47) (available for *n* = 12)	0.44 (± 0.17) (available for *n* = 9)	0.48 (± 0.17) (available for *n* = 9)	1.0 (1.0) (available for *n* = 16)	11/13 (84.6)	2/13 (15.4)	0	0	0	NA	3.84 (± 3.95) (available for *n* = 13)
CIS *n* = 1	0 (0)	51.64	NA	NA	NA	NA	1.0 (0)	NA	NA	NA	NA	NA	2.06	NA
ON *n* = 2	1 (50.0)	36.56 (± 5.87)	0.9 (± 0.14)	1.13 (± 0.18)	0.13 (available for *n* = 1)	0.2 (available for *n* = 1)	1.0 (0) (available for *n* = 1)	1/1 (100.0)	0	0	0	0	2.58 (± 0.23)	NA
RRMS *n* = 184	131 (71.2)	41.26 (± 11.92)	0.96 (± 0.32) (available for *n* = 130)	0.97 (± 0.31) (available for *n* = 131)	0.32 (± 0.18) (available for *n* = 93)	0.3 (± 0.17) (available for *n* = 96)	2.0 (1.5) (available for *n* = 174)	95/163 (58.3)	42/163 (25.8)	19/163 (11.7)	5/163 (3.1)	2/163 (1.2)	11.66 (± 9.66) (available for *n* = 174)	9.98 (± 9.04) (available for *n* = 182)
SPMS *n* = 20	13 (65.0)	54.35 (± 11.68)	0.72 (± 0.28) (available for *n* = 12)	0.82 (± 0.36) (available for *n* = 13)	0.22 (± 0.07) (available for *n* = 11)	0.18 (± 0.11) (available for *n* = 11)	5.25 (2.5)	6/17 (35.3)	2/17 (11.8)	5/17 (29.4)	4/17 (23.5)	0	20.75 (± 8.51) (available for *n* = 18)	19.28 (± 8.87)
PPMS *n* = 18	9 (50.0)	54.96 (± 10.4)	0.88 (± 0.22) (available for *n* = 12)	0.81 (± 0.27) (available for *n* = 12)	0.25 (± 0.14) (available for *n* = 6)	0.16 (± 0.14) (available for *n* = 6)	3.75 (2.125)	8/15 (53.3)	6/15 (40.0)	1/15 (6.7)	0	0	11.04 (± 10.5)	8.62 (± 9.52)
NMOSD *n* = 16	12 (75.0)	50.87 (± 12.99)	0.67 (± 0.3) (available for *n* = 11)	0.76 (± 0.26) (available for *n* = 11)	0.18 (± 0.15) (available for *n* = 12)	0.23 (± 0.13) (available for *n* = 12)	4.0 (4.375) (available for *n* = 8)	3/5 (60.0)	0	2/5 (40.0)	0	0	10.39 (± 9.19) (available for *n* = 14)	7.95 (± 8.1) (available for *n* = 13)
MOGAD *n* = 14	11 (78.6)	36.11 (± 15.1)	0.88 (± 0.35) (available for *n* = 13)	0.71 (± 0.35) (available for *n* = 13)	0.19 (± 0.1) (available for *n* = 12)	0.18 (± 0.15) (available for *n* = 12)	2.0 (4.25) (available for *n* = 8)	3/5 (60.0)	1/5 (20.0)	1/5 (20.0)	0	0	4.57 (± 5.19) (available for *n* = 12)	4.36 (± 5.0) (available for *n* = 12)
Immune-mediated peripheral neuropathy *n* = 19	6 (68.4)	61.51 (± 13.64)	0.55 (± 0.35) (available for *n* = 2)	0.8 (available for *n* = 1)	0.25 (available for *n* = 1)	0.4 (available for *n* = 1)							9.26 (± 8.66) (available for *n* = 5)	7.83 (± 9.07) (available for *n* = 5)
Myasthenia gravis *n* = 10	7 (70.0)	41.32 (± 12.18)	1.02 (± 0.37) (available for *n* = 9)	0.97 (± 0.39) (available for *n* = 8)	0.3 (± 0.2) (available for *n* = 7)	0.3 (± 0.22) (available for *n* = 7)							8.7 (± 8.16) (available for *n* = 9)	7.69 (± 8.44) (available for *n* = 9)
Overlap syndrome *n* = 3	3 (100.0)	62.87 (± 6.03)	NA	NA	NA	NA							16.08 (available for *n* = 1)	16.08 (available for *n* = 1)
Huntington’s disease *n* = 33 (*n* = 20 manifest, *n* = 13 premanifest)	21 (63.6)	46.45 (± 15.21)	0.87 (± 0.66) (available for *n* = 2)	0.92 (± 0.59) (available for *n* = 2)	0.2 (available for *n* = 1)	0.16 (available for *n* = 1)							5.00 (± 3.45) (only for manifest HD, *n* = 20)	NA
Post-COVID *n* = 2	1 (50.0)	40.33 (± 28.68)	1.25 (available for *n* = 1)	0.4 (available for *n* = 1)	1.0 (available for *n* = 1)	0.4 (available for *n* = 1)							2.13 (available for *n* = 1)	NA
Other neurological diseases *n* = 7	3 (42.9)	48.56 (± 15.69)	0.8 (available for *n* = 1)	1.0 (available for *n* = 1)	0.25 (available for *n* = 1)	0.32 (available for *n* = 1)							5.14 (± 7.84) (available for *n* = 4)	8.41 (± 11.89) (available for *n* = 2)

*CIS* Clinically Isolated Syndrome, *COVID* Coronavirus disease, *EDSS* Expanded disability status scale, *FS* Functional score, *HCVA* High-contrast visual acuity, *IQR* Interquartile range, *LCVA* Low contrast visual acuity, *MOGAD* Myelin Oligodendrocyte Glycoprotein Antibody-Associated Disease, *MS* Multiple Sclerosis, *NA* not available, *NMOSD* Neuromyelitis Optica spectrum disorder, *ON* isolated optic neuritis, *PPMS* Primary Progressive Multiple Sclerosis, *RIS* Radiologically Isolated Syndrome, *RRMS* Relapsing–Remitting Multiple Sclerosis, *SD* Standard deviation, *SPMS* Secondary Progressive Multiple Sclerosis

Within the ND group, 243 were MS-spectrum diseases (*n* = 18 Radiologically Isolated Syndrome [RIS], *n* = 1 Clinically Isolated Syndrome [CIS], *n* = 184 Relapsing Remitting MS [RRMS], *n* = 20 Secondary Progressive MS [SPMS], *n* = 18 Primary Progressive MS [PPMS], *n* = 2 isolated optic neuritis [ON]) with a median EDSS of 2.0 (IQR 0–4.0) and ranging from 0 to 8.5 and a mean disease duration of 11.85 (SD ± 9.85) years. In MS, 164 were female (67.5%). High-contrast visual acuity was well preserved within the MS group as compared to HC (Table 1).

The cohort of other ND consists of neuromyelitis optica spectrum disorder (NMOSD, *n* = 16; aquaporin-4 (AQP4-) antibody positive, *n* = 12, negative, *n* = 2, unknown, *n* = 2), myelin oligodendrocyte glycoprotein antibody-associated disorder (MOGAD, *n* = 14, all MOG-antibody positive), immune-mediated peripheral neuropathy (*n* = 19), myasthenia gravis (MG, *n* = 10), overlap syndrome (*n* = 3), HD (*n* = 33), Post-COVID syndrome (*n* = 2), and other ND (*n* = 7) (Table 1).

### Detection and prevalence of INO

Review of the VR-based oculography reports revealed 43 cases of INO (*n* = 3 HC, no bilateral INO; *n* = 40 ND, *n* = 26 unilateral, *n* = 14 bilateral). For those with corresponding clinical data, we observed moderate agreement of VR-based oculography and clinical observation (Cohen’s Kappa 0.42, 92.5% observer agreement, available for *n* = 414 due to missing data for the clinical assessment, Table 2). There were 28 cases detected by VR-based oculography only and 3 cases only clinically, 370 congruent cases without and 13 congruent cases with INO, respectively.

**Table 2:** Prevalence of internuclear ophthalmoplegia based on the VR-oculography and the clinical evaluation for each group

	VR-based INO, *n* (%)	Clinical examination INO, *n* (%)
Healthy persons	3/124 (2.4)	0/116 (0.0)*
MS-spectrum diseases	37/243 (15.2)	16/238 (6.7)^#^
RIS	0/18 (0.0)	0/18 (0.0)
CIS	0/1 (0.0)	NA
ON	0/2 (0.0)	0/2 (0.0)
RRMS	27/184 (14.7)	10/181 (5.5)^#^
SPMS	8/20 (40.0)	5/19 (26.3)
PPMS	2/18 (11.1)	1/18 (5.6)
NMOSD	1/16 (6.3)	0/12 (0.0)^#^
MOGAD	1/14 (7.1)	0/11 (0.0)
Immune-mediated peripheral neuropathy	1/19 (5.3)	0/7 (0.0)
Myasthenia gravis	0/10 (0.0)	0/10 (0.0)
Overlap syndrome	0/3 (0.0)	0/3 (0.0)
Huntington’s disease	0/33 (0.0)	0/8 (0.0)
Post-COVID	0/2 (0.0)	0/2 (0.0)
Other neurological disorders	0/7 (0.0)	0/7 (0.0)

*COVID* Coronavirus disease, INO Internuclear ophthalmoplegia, *MOGAD* Myelin Oligodendrocyte Glycoprotein Antibody-Associated Disease, *MS* Multiple sclerosis, *NA* not available, *NMOSD* Neuromyelitis Optica spectrum disorder, *VR* Virtual reality

*Clinical data was present for all three healthy persons with VR-based INO

^#^Clinical data was missing for *n* = 1 with VR-based INO

INO cases in HC presented as a relative unilateral adduction deficit with considerably higher absolute saccadic velocities than in INOs seen in MS cases in the first HC. In the second HC case, the eye-tracking was complicated by drooping eyelids, technical reasons might thus play a role in this case, yet it might represent a subclinical finding. In the last case, no artifact sources could be identified in the video file, thus fulfilling our definition of INO. It may represent Duane syndrome (congenital and asymptomatic). Aggregated data of these participants are given in Table 3.

**Table 3:** Characteristics of study participants with and without internuclear ophthalmoplegia based on the VR-oculography for multiple sclerosis and healthy controls

	MS-spectrum diseases *n* = 243			Healthy control *n* = 124		
	With VR-based INO *n* = 37	Without VR-based INO *n* = 206	*p* value	With VR-based INO *n* = 3	Without VR-based INO *n* = 121	*p* value
Sex, female, *n* (%)	19 (51.4)	145 (70.4)	0.035	1 (33.3)	70 (57.9)	0.575
Age, y, mean (SD)	48.73 (± 11.59)	41.65 (± 12.64)	0.002	65.01 (± 9.68)	41.90 (± 16.2)	0.014
Time from onset, mean (SD)	16.6 (± 10.04) (available for *n* = 34)	11.0 (± 9.6) (available for *n* = 191)	0.002			
Time since diagnosis, mean (SD)	14.31 (± 8.99)	9.58 (± 9.24) (available for *n* = 196)	0.002			
EDSS, median (IQR)	3.5 (2.5)	2.0 (1.9) (available for *n* = 192)	< 0.001			

*EDSS* Expanded disability status scale, *INO* Internuclear ophthalmoplegia, *IQR* Interquartile range, *MS* Multiple sclerosis, *SD* Standard deviation, *VR* Virtual reality, *y* year

Statistics: Fisher’s exact test for comparison of sexes, all others Mann–Whitney *U* test

Focusing ND, the VR-based oculography tool detected INO in 37/243 MS (15.2%), 1/16 NMOSD (6.3%), 1/14 MOGAD (7.1%), 1/19 immune-mediated peripheral neuropathy (5.3%, anti-GQ1b negative), 0/10 MG, 0/3 Overlap syndrome, 0/33 HD, 0/2 Post-COVID syndrome, and 0/6 other ND participants (Table 2). In the patient with immune-mediated peripheral neuropathy, gaze holding, ocular alignment, and smooth pursuit were not indicative of third nerve palsy, and again, absolute saccadic velocities were within the normal range as defined by the main sequence. The latter holds true for the MOGAD patient, respectively. The NMOSD patient (AQP4 antibody positive) was also within the normal range; however, compared to the contralateral saccadic velocities, both ad- and abduction were diminished with relative adduction slowing fulfilling our INO definition.

### Demographic characteristics of MS patients with and without INO

Presence of INO measured by the VR-based oculography tool was highest in SPMS, occurring in 40.0% of patients (8/20), followed by RRMS with 14.7% (27/184) and PPMS with 11.1% (2/18). Within the MS cohort without INO in the VR-based assessment, 70.4% were female (145/206). Mean age of this cohort was 41.65 ± 12.64 years. Of the 37 patients with MS and INO in the VR-based assessment, 18 were male (48.7%) and 19 were female (51.4%). The mean age was 48.73 ± 11.59 years (Table 3).

Detection of INO with the VR-based oculography tool was significantly more frequent in male sex (*p* = 0.035), with higher total EDSS and higher brainstem FS score (all *p* < 0.001), longer time from onset, from diagnosis and higher age (all *p* = 0.002, Table 3). Presence of subjective visual symptoms was also associated with detection of INO (*p* = 0.007).

**Fig. 3:**
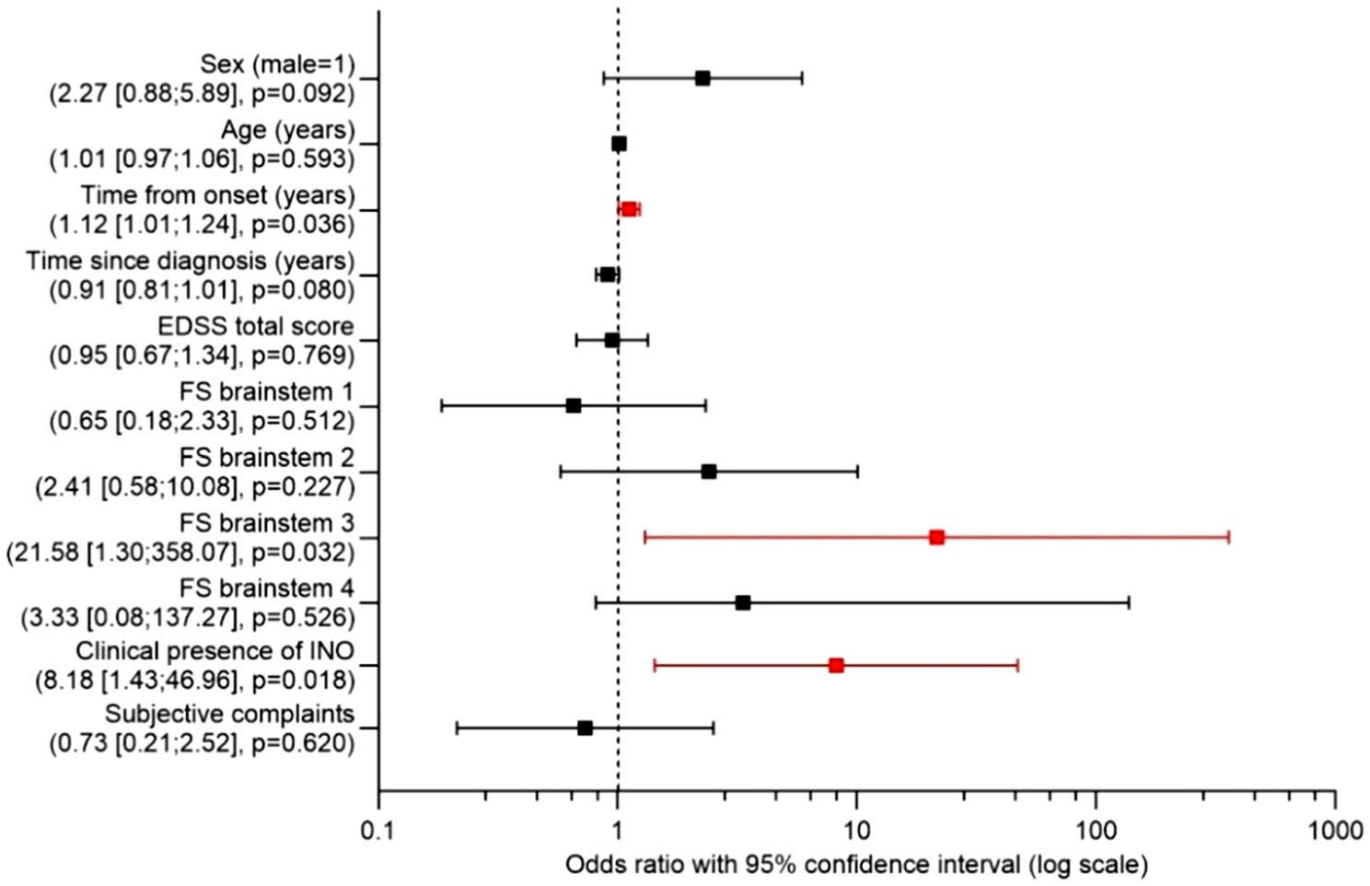
Binary logistic regression model of factors associated with the presence of INO detected by VR-based oculography (dependent variable) within the MS cohort. Odds ratios (OR) with 95% confidence intervals on a logarithmic scale are displayed. Independent variables included in the model: sex (ref. male = 1), age (years), time from disease onset (years), time since diagnosis (years), Expanded disability status scale (EDSS) total score, functional system (FS) brainstem subscores 1–4 (ref. FS 0), clinical presence of INO, and subjective visual complaints. Significant associations are marked in red. ORs greater than 1 indicate a positive association between the respective independent variable and the presence of VR-based INO (higher odds for presence of VR-based INO), ORs below 1 indicate a negative association thereof (lower odds of VR-based INO), respectively

Consecutively, the binary logistic regression model included age, sex, time from onset, from diagnosis, total EDSS, brainstem affection in EDSS, clinical presence of INO, and presence of subjective symptoms as independent variables and presence of INO in the VR-based assessment as dependent variable. It demonstrated significant associations with time from onset, higher brainstem FS score and clinical presence of INO (Fig. 3).

Within the MS cohort, a moderate agreement of VR-based oculography and clinical observation was likewise detected (Cohen’s Kappa 0.45, 89.1% observer agreement, available for *n* = 238 due to missing data for the clinical assessment, Table 2). There were 23 cases identified by the novel oculography tool alone and three cases identified by clinical examination only, with 199 congruent negative and 13 congruent positive INO cases, respectively.

### Evaluation of corresponding INO lesions in available routine brain MRI scans

For the total cohort of 46 INO cases (43 VR-based with or without clinical INO and 3 only clinical INO), no brain MRI was available for *n* = 10 (21.7%) including the three HC. Another 10 brain MRI scans were lacking sufficient images over the brainstem/MLF (21.7%). Of remaining 26 scans, a corresponding MLF lesion could be identified in 22/26 cases (84.6%).

Corresponding MRI and VR findings were present in 20/22 cases. Agreement of all three measures (MRI, VR, clinical) was detected in 9 cases, 11 cases demonstrated an MRI and VR finding, but were lacking the clinical correlate of INO.

Only 2 cases demonstrated an MRI and clinical lesion without a VR correlate (P(MRI + │VR-) = 2/2).

All 4 cases without an MRI lesion were solely VR-based INO findings.

Conditional probabilities for a positive MRI finding in case of a positive VR result (P(MRI + │VR +)) was 20/24 (83.3%) and in case of a positive clinical result (P(MRI + │clinical +)) 11/11 (100.0%), respectively. However, of 15 negative clinical findings, 11 cases had a positive MRI (P(MRI + │clinical-), 73.3%).

## Discussion

We demonstrate the implementation of a novel VR-based tool in daily clinical routine. With only a few terminated examinations, the method was generally well tolerated and feasible even in severely disabled patients.

With the high agreement rates for the total (92.5%) and the MS cohort (89.1%), reliability of the method was demonstrated in both cohorts. Given the absence of an independent reference standard for INO, agreement statistics were considered more appropriate than sensitivity and specificity, which would have implied hierarchical assumptions regarding the clinical examination.

We hypothesize that the VR-based oculography enables more precise assessment of eye movement disorders, in particular INO, as the VR-based oculography tool detected more INOs than clinical examination. Visual observation of eye movements seems to be inferior to the automated assessment as the latter uses repeatedly measured saccadic peak velocities. It seems plausible that the automated detection captures subtle neurological dysfunction at a lower threshold as visual inspection alone. Reliable visual observation of eye movements requires specialist training and expertise. Mild INO can easily be overlooked [13], as it depends on subjective evaluation and examination environment [20]. False-positive findings by the novel VR-based oculography tool cannot be fully excluded. However, corresponding MLF lesions in the MRI scans were detectable in the large majority of VR-based INO cases (83.3%), irrespective of the clinical finding, whereas a considerable proportion of cases without clinical INO presented with a corresponding MRI lesion (73.3%). However, imaging data were only available in a small subset of patients and derived from available routine MRI, thereby limiting the validation. We may not securely decipher whether the four solely VR-positive findings represent overreading or a true functional deficit not reflected in routine brain MRI [6]. This MRI paradox has been framed before for infrared oculography in MS describing the discrepancy between the evidence of INO in functional metrics and absence of a neuroanatomical correlate using MRI [21]. Advanced MRI techniques such as diffusion tensor imaging (DTI) or ultra-high field MRI may help to overcome the lower detection rates for MLF lesions [22] which were not available in this pilot study.

Three cases of INO were detected by our visual report-based blinded assessment in HC. This is a limited number; however, they fulfilled our definition of INO and only one case might have been explained by artifacts of the measurement. A small case series of pseudo-INO in MG reported a phenomenon that we observed in one participant, likewise, with a difference of saccadic ad- and abduction velocities, but clearly higher absolute velocities as compared to an MS group [23]. The integration of absolute peak velocities into any INO algorithm might thus be valuable to better differentiate from pseudo-INO. As the HC group did not undergo additional testing (e.g., MRI, autoantibody or electrophysiological screening), we cannot elaborate on potential subclinical or early disease not yet diagnosed. Whether VR-based oculometry might also serve as a screening tool in neurological disease needs specifically designed studies. A potential contribution of age should be assessed therein as our three participants were older than HC without INO.

Fatigue represents an influencing factor of saccadic peak velocities, both in healthy and MS populations [24, 25]. It has been described to influence task performance with increasing age. However, task duration was considerably longer (40 min versus less than 10 min in our study) [24]. It can be assumed that fatigue results in symmetric slowing, especially in healthy subjects without a known underlying disease affecting ocular motor function. As we did not evaluate fatigue in our setting, an influence may not be fully excluded. Yet, mimicking of pseudo-INO with differences of ad- and abducting saccade peak velocities does not seem likely, especially for HC.

In the cohort of immune-mediated peripheral neuropathy, there was one case of INO without signs of third nerve palsy and with a mild result as visually compared to those of patients with MS. Central manifestation is rare, but described predominantly in the presence of anti-GQ1b antibodies which were not present in our case [26]. In MG, overlap syndrome, HD, Post-COVID syndrome, and other ND, there were no cases of INO. INO can occur in the course of these diseases. Yet, it would be an uncommon manifestation or rather a mimicry of INO (pseudo-INO) that may be more clearly dissected using technical methods than clinical examination alone [27, 28]. Abnormal saccadic eye movements are a well-known feature in HD [29], but was likewise not mistaken for INO using our VR-based assessment.

Prevalence of INO was highest in MS. Presence of INO was more than twice as common in MS than in NMOSD. This circumstance may facilitate the differentiation between MS and NMOSD as described earlier [12]. In MOGAD, INO is described in exceptional cases only, corresponding to our results and representing a mild finding in visual comparison to INO cases in MS [30]. Our findings suggest that VR-based assessment may detect ocular motor abnormalities compatible with INO not only in MS but also across a broader spectrum of neurological diseases. This supports the potential utility of the tool as a disease-independent functional screening method for MLF-related ocular motor dysfunction, but due to the rarity of the respective diseases also requires further investigation to assess its diagnostic validity.

The sex ratio of our MS cohort with a female-to-male ratio of 2.1:1 resembles the demographic distribution of MS [31]. INO prevalence in our MS cohort was 1.97 times higher in men than in women with a significant difference tested as a single factor, yet, not confirmed as a major contributor in the regression model. Potential sex-specific patterns of lesion distribution thus need further investigation. Indeed, within MSBase, brainstem involvement was more common in men in these observational data lacking corresponding imaging data [32]. However, other data did not support consistent evidence of greater brainstem involvement in men with MS, as determined by MRI and constellation of symptoms [33, 34]. Sex-related information about eye movement abnormalities in MS is thus still sparse and partly inconsistent.

In our MS cohort, the presence of INO was associated with higher total EDSS and higher brainstem FS score, longer time from onset, from diagnosis and higher age. Correspondingly, prevalence was highest in SPMS. In the final regression model, time from onset, more severe brainstem involvement and clinical presence of INO remained significant factors for INO detection using VR technology. A correlation has been described between INO and higher EDSS scores, as well as between INO and higher brainstem FS Scores [1, 21]. This is generally plausible as INO, reflecting MLF lesions, is part of the brainstem assessment within the EDSS. However, numerous other findings are summarized in the brainstem FS score [9]. Although INO can occur at any stage of disease, its presence was reported to be more common in patients with longer disease duration [1, 21] as in our cohort. Still, the presence of INO should not be used as an indicator for disease duration, since accumulation of disability increases over time, but not all patients develop brainstem involvement and some may do early in their disease course. INO seems to be an age-independent, but common manifestation of brainstem involvement [4, 35]. Although increasing age correlates with lower relapse rates, disability accumulates faster, yet, not necessarily by occurrence of INO [36, 37].

The binary logistic regression model identified time from onset, higher FS brainstem score, and clinical presence of INO as significant factors associated with presence of INO using VR-based oculography. Except for the lacking association of the total EDSS in this model, these findings confirm the previous studies based on clinical data [9, 32–34] and on an infrared oculography protocol [1, 21], using our novel approach for VR-based detection of INO based on simple visual assessment.

A recent study has introduced a specific infrared oculography protocol and algorithm to provide an objective and quantitative approach to diagnosing INO [1]. Participants had to maintain a specific position, while following instructions displayed on a screen, while being tracked by infrared cameras. The eye movement data were converted into the versional dysconjugacy index (VDI), describing the ratio between the abducting and adducting eye. The index facilitates pattern recognition and identification of INO. Another study has applied this protocol and method, underlining its reliability [3]. They reported a potential insufficient specificity for the VDI thresholds, detecting INO in 17.3% (19/110) of healthy controls [3]. The setting of thresholds may thus be adapted to the specific research question or clinical application. Another study investigated the VDI by applying the calculation to data obtained using a video-oculography tool (VOG) [6]. During the examination, the participants wore the VOG tool and sat at a specific distance from the screen displaying the instructions [6]. As compared to these repeatedly confirmed elaborated setups, our approach aims to establish a method for easy visual identification of INO that may be applied in various clinical routine settings. Within our pilot setup, this was performed in a blinded manner by two to three independent raters. However, compared to automated algorithms, this may introduce a potential bias for subjectivity which will be even more present in unblinded clinical routine conditions so that again, maybe a combination of the two approaches will be the best future direction. The setup allows for testing in a variety of positions, enabling examination on severely disabled or bedridden patients using a headset and mobile system.

There are different systems available to perform advanced eye tracking, e.g., EyeLink devices (https://www.sr-research.com/) or devices by Tobii (https://www.tobii.com/de). However, research areas of published applications are wide, but mostly not comparable to our basic clinical approach. We have only identified a single study (*n* = 5 MS, *n* = 17 HC) using one of these devices to assess saccades in INO [38] and did not perform a direct comparison of devices ourselves.

The observed associations to clinical parameters support the construct validity of the VR-derived measures and corroborate previous findings. If further substantiated in external settings, VR-based oculography may provide a functional readout of brainstem involvement with clinical relevance to MS and other conditions. The VR-based assessment provides quantitative and reproducible metrics [14] making it an attractive tool for early detection of brainstem dysfunction, but also for longitudinal follow-up potentially suitable for quantification of treatment response [39] or even as an objective functional outcome parameter in clinical trials. It may even be used in telemedicine supported care pathways lowering barriers for a detailed neuroophthalmic work-up. A comparatively low level of training is required for the correct use of the device. However, costs need to be taken into account when addressing the question of better access to care.

### Strengths and limitations

The standardized conduction of the VR-based examination is a clear advantage over the clinical observation alone. Our aim was to explore a visual approach for determining INO based on that standardized examination that may easily be transferred to clinical applications. To address the bias introduced by this visual interpretation, we used a blinded rating by two to three independent raters. However, automated quantitative measures such as VDI might still be more objective, but have mostly failed to reach clinical routine thus far. The association of VR-detected INO to assumingly less granular measures such as brainstem involvement by EDSS and agreement with clinical INO makes the results generally plausible and robust. Yet, the assumption that the VR-based assessment reliably detects additional cases of INO missed during clinical examination should be explored further, for instance using advanced MRI techniques as MRI was not a focus of this pilot study and only available for a subset of participants. Our study is limited by its monocentric design and the inherent limitation regarding the diversity of our cohort. Preexisting visual or ocular motor abnormalities did not serve as an exclusion criterion as we deemed this to introduce additional selection bias. However, INO detection might be obscured in some cases by this approach. An external validation will be essential to determine generalizability to other settings and clinical utility. Although our sample size is comparatively large, independent confirmation will particularly be relevant for the rarer conditions such as NMOSD and MOGAD. Future studies should also investigate potential confounding factors such as fatigue in neurological conditions, but also in healthy populations.

## Conclusion

If further substantiated in independent and diverse settings, VR-based eye-tracking metrics may serve as complementary digital biomarkers of brainstem dysfunction with diagnostic and monitoring potential in different neurological disorders. Our investigations in a large MS cohort showed moderate agreement with clinical assessment, but identified additional cases of INO associated with markers of disease burden. This study employed an easy-to-use visual approach close to the clinics. However, for different research questions, quantitative metrics may be better suited if suited thresholds respecting sensitivity and specificity within a specific research application are established. Immersive VR-based eye-tracking may alleviate detection of subtle neurological dysfunction not captured by ordinal bedside testing or routine imaging and thus help to identify progression of disease, especially in long-term follow-up. Its potential as a screening tool in populations at risk for different conditions and its use as an objective outcome biomarker in clinical trials should be further explored.

## Supplementary Information

Below is the link to the electronic supplementary material.

Supplementary file1 (DOCX 32 KB)
