# Supplementary material for: Virtual reality-based oculography detects internuclear ophthalmoplegia in multiple sclerosis and other neurological disorders
Source: J Neurol. 2026 Jun 6;273(7):372. doi: 10.1007/s00415-026-13906-x (PMC13242395; doi:10.1007/s00415-026-13906-x)
Supplement: Supplementary file 1 — Supplementary file1 (DOCX 32 KB) [file 415_2026_13906_MOESM1_ESM.docx]

**Supplementary Methods**

**Technical details of the Virtual-reality (VR-) based oculography device**

**Manufacturer’s details:**

machine**MD** AG
Weyermannsstrasse 36

3008 Bern

Switzerland

<https://www.machinemd.com/contact>

**Device details:**

ne**o**s^™^, is certified as a Class IIa medical device under the European Union Medical Device Regulation (EU-2017/745 MDR) by TÜV SÜD Danmark (NB 2443).

**Measurement details:**

The VR-based oculography system generates visual stimuli on the LED display of the Varjo VR headset.

Eye and pupil movements are recorded using an integrated infrared sensor operating at a frequency of 200 Hz. If needed, corrective lenses can be inserted into the headset to adjust for refractive errors ranging from −6 to +4 diopters. Inter-pupillary distance (IPD) is mechanically adjusted in the headset.

No special corrections are applied for headset slippage. Due to the head centric presentation, isolated eye movements are recorded independently of head movements.

Calibration is performed monocularly for each eye.

The examination protocol consists of five consecutive stimulus blocks. The full assessment takes approximately 10–12 minutes. Individual blocks can be selected as needed. Each block is defined by the specific type of stimulus presented, and multiple parameters may be assessed within a single block. The task conduction is mostly intuitive. However, in our setting, a short verbal instruction is given with the start of each examination block. No practice trials have been performed with the participants.

Horizontal saccadic eye movements are evaluated in the "Eye Movement" block, which features a stimulus in the form of a fairy with a glowing circle that rapidly shifts position - five times between ±10° and four times between ±20° along the horizontal axis. The stimulus is always presented to both eyes, i.e. binocularly.

The stimulus presentation includes fixation for one second and no gap between stimuli. The stimulus is presented in a pseudo-randomized order of direction and amplitude.

Valid saccadic peak velocities are grouped into amplitude intervals of 7.5°–12.5° (centered on 10°) and 17.5°–22.5° (centered on 20°), each spanning 5°. Using 5° bins offers a practical balance between spatial resolution and statistical power and ensures that saccades at clinically relevant amplitudes (10°, and 20°) are captured accurately, supporting reliable comparisons across studies and clinical assessments. This approach has been described earlier [1].

For each bin, saccades are aggregated to compute a weighted mean peak velocity, its standard deviation, as well as the mean amplitude and its standard deviation. The weighting is based on the inverse squared uncertainty of each individual saccade, ensuring that more reliable measurements have a stronger influence on the final estimate.

A test-retest trial of the device has been performed to determine reliability and precision of gaze assessment [2].

Upon completion of the examination, the software automatically generates a report containing the recorded data.

**Additional references**

1. Hopf S, Liesenfeld M, Schmidtmann I et al. (2018) Age dependent normative data of vertical and horizontal reflexive saccades. PLoS ONE 13(9): e0204008.

doi: 10.1371/journal.pone.0204008

1. Coito A, Naidu A, Lehmann J et al. (2025) Test-retest reliability of gaze precision of a novel virtual reality-based medical device. Front. Virtual Real. 6:1502679.

doi: 10.3389/frvir.2025.1502679
